# Exploring the health benefits of raw white garlic consumption in humans: a mini review

**DOI:** 10.3389/fnut.2024.1459627

**Published:** 2024-08-30

**Authors:** Rebeka Fejes, Catherine P. Bondonno, Simone Radavelli-Bagatini, Tilman Kühn, Karl-Heinz Wagner

**Affiliations:** ^1^Department of Nutritional Sciences, University of Vienna, Vienna, Austria; ^2^Vienna Doctoral School of Pharmaceutical, Nutritional and Sport Sciences, University of Vienna, Vienna, Austria; ^3^Nutrition and Health Innovation Research Institute, School of Medical and Health Sciences, Edith Cowan University, Joondalup, WA, Australia; ^4^Centre for Public Health, Medical University of Vienna, Vienna, Austria

**Keywords:** allicin, *Allium sativum*, cancer, cholesterol, garlic, human health

## Abstract

**Introduction:**

Raw white garlic, a fundamental food in both culinary and medicinal practices globally, has gained attention for its potential health benefits. Despite widespread use, clinical research has predominantly focused on aged black garlic or garlic extracts, leaving raw white garlic consumption in humans underexplored. This mini review aims to summarize the evidence from clinical and observational studies on the health effects of raw white garlic consumption.

**Methods:**

A search in PubMed and Scopus databases was conducted to identify clinical and observational studies on raw white garlic consumption. Twelve clinical trials and 10 observational studies meeting the predefined inclusion criteria were selected for review.

**Results:**

Results from clinical trials revealed diverse health effects of raw garlic consumption, including improved lipid profiles, blood pressure regulation, fibrinolytic activity, antioxidant status, and glucose metabolism. Observational studies reported the association of raw garlic consumption with improvements of important health outcomes, including cancer risk, cardiovascular disease, insulin homeostasis, and liver function. However, both clinical and observational studies were heterogenous in design, participant characteristics, durations, and outcome measures. Observational studies were limited to Asian populations.

**Conclusion:**

While human studies indicate that raw garlic may exert various health benefits, larger randomized controlled trials with longer follow-up and cohort studies are needed to explore the full potential of raw garlic consumption in human health promotion. Our mini-review aims to summarize the currently available evidence on raw garlic consumption in humans.

## 1 Introduction

Meta-analyses show lipid-lowering nutraceuticals like garlic, red yeast rice, plant sterols and further similar compounds are effective and safe, with no major safety issues have been found (1). They may improve lipid levels in those with mild-to-moderate dyslipidemia and low cardiovascular risk (1).

Raw white garlic has been a culinary and medicinal pillar across cultures (2), and its potential health benefits have intrigued researchers. However, most clinical studies exploring the health effects of garlic on humans have predominantly focused on aged black garlic or garlic extracts (3, 4)—often as proprietary formulations and sold as expensive dietary supplements. While these studies provide valuable insights into the potential health effects of garlic compounds, their extrapolation to raw white garlic is limited due to the altered chemical composition resulting from the aging process or extraction methods (5). Various drying methods employed in industry to prepare different garlic products result in decreased levels of bioactive constituents such as allicin, total phenols, and pyruvate compared to freshly harvested garlic (5). Instead, they contain various products resulting from allicin transformation. For example, sulfur compounds in fresh garlic may be nearly 1,000 times more potent antioxidants compared to those in aged garlic extract (6). The inconsistencies between research focused on garlic supplements versus traditional raw white garlic consumption worldwide highlights the need for comprehensive investigations into the health benefits and mechanisms of action of raw garlic. Future research could provide a holistic understanding of the potential benefits associated with the consumption of raw garlic. Therefore, the aim of this mini review is to explore current evidence on the potential benefits of raw garlic consumption from clinical and observational studies conducted in humans.

## 2 Methods

PubMed and Scopus electronic databases were used for literature search using the following search terms: (“raw garlic” OR “white garlic”) without limiting the publication dates. Only studies with human participants published in English were extracted. Only trials that used raw white garlic as the intervention, or observational studies examining the associations of consumption of raw white garlic with health benefits were included. Studies involving other garlic preparations such as dried garlic, garlic oil or juice extracted from raw crushed garlic were excluded. The initial search hits were cleared from duplicates and subsequent screened, leading to the selection of 12 clinical trials (7–18) and 10 observational (19–28) studies.

## 3 Clinical trials

Details of the twelve clinical trials included, in which seven were randomized controlled trials (RCTs) (8, 9, 11, 14–17), are summarized in Table 1. Number of participants varied from 5 to 49 and the age of participants ranged between 17 and 70 years of age [only the mean age was available in 4 studies age (11, 14–16)]. Six studies (8, 14–18) included both female and male participants. Eight out of 12 studies reported favorable impacts on human health following the consumption of fresh garlic (7–13, 18), three showed no effects (14, 15, 17), and one provided exploratory data (16) For one publication (12), only the abstract was available. The health benefits included improved blood lipid levels (7–10, 12, 18), reduced systolic and diastolic blood pressure (8, 18), as well as enhancements in fibrinolytic activity (8, 10, 11), higher superoxide dismutase (SOD), catalase (CAT) and glutathione peroxidase activity (GPx) (7), reduced benzo[a]pyrene-DNA adduct levels (13), decreased fasting blood glucose concentration (7, 12, 18), reduced waist-hip ratio (18) and body mass index (BMI) (8). Regarding lipid levels, five studies showed reductions in total/serum cholesterol levels (7–10, 12), four showed decreased triglyceride (TG) concentrations (7, 8, 12, 18), two trials found low-density lipoprotein (LDL)-lowering effects (7, 8) and four studies reported an increase in high-density lipoprotein (HDL) cholesterol (7, 8, 12, 18). Furthermore, 4.2 g/day of raw garlic supplementation did not impair platelet function (15) and 5 g/day resulted in gene upregulation related to immunity, apoptosis and xenobiotic metabolism (16). Six studies were conducted on healthy subjects (9, 10, 13–16), while three investigated participants with a precondition such as metabolic syndrome (18), ischemic heart disease (11), (moderate) hyperlipidemia (8, 12, 17) and diabetes mellitus type 2 (7). Nevertheless, seven clinical studies (7, 8, 12, 15–18) were conducted between 2006 and 2018, while five (9–11, 13, 14) were published between 1979 and 1999, with the outcome variables exhibiting considerable diversity. The duration of the study interventions varied from a single dose of garlic (11, 15, 16) to 6 months (17) and the dosage ranged from 4 g on 6 days of the week to 20 g daily and 0.1 to 0.5 g per kg bodyweight per day (KG BW/d) (e.g., 35 g/day for a 70 kg person), respectively. Additionally, two trials also investigated acute effects of 5 h (15), 6 h and 12 h (11) after raw garlic consumption. Platelet function was not impaired 5 h after 4.2 g raw garlic ingestion (15) and the fibrinolytic activity increased after 6 h and was maintained until 12 h post consumption (11).

**Table 1:** Clinical studies investigating the effects of raw garlic on human health.

Reference	Sample size	Age (years)	Sex	Dose	Duration	Outcome	Results
Choudhary et al. (18)—India	40	30–70	m/f	2 × 100 mg/KG BW/d	4 weeks	Components of metabolic syndrome	↓ SBP, DBP, TG, FBG, WC ↑ HDL ↔ BMI
Charron et al. (16)—United States	17	54.9 (mean)	m/f	5 g/d	Single dose	Expression of immunity- and cancer-related genes	↑ AHR, ARNT, HIF1A, JUN, NFAM1, OSM, REL
Scharbert et al. (15)—Austria	18	29 (mean)	m/f	4.2 g/d	Single dose	Platelet function (acute)	↔ ASPI- and COLL-aggregation, PFA-closure time, MA-reduction
	5		Not reported	4.2 g/d	1 week	Platelet function (long-term)	
Gardner et al. (17)—United States	RG = 49, garlicin = 47, kyolic = 48, placebo = 48	30–65	m/f	4.0 g, 6 d/week	6 months	Plasma lipid concentrations	↔ LDL, HDL, TC:HDL, TG
Mahmoodi et al. (12)	30	Not reported	NA	2 × 5 g/d	6 weeks	Blood biochemical factors	↓ TC, TG, FBG ↑ HDL
Munday et al. (14)—New Zealand	RG = 9, control = 27	36.6 (mean)	m/f	6 g/d	7 days	Resistance to *in vitro* Cu^2+^-mediated LDL oxidation	↔ Resistance to *in vitro* Cu^2+^-mediated LDL oxidation, TC, TG, HDL, HDL:TC
Hageman et al. (13)—The Netherlands	9	Not reported	m	3 g/d	8 days	Anticarcinogenic potentioal	↓ BP-DNA adduct ↑ NAT activity
Gadkari and Joshi (10)—India	RG = 30, control = 20	17–22	Not reported	10 g/d	2 months	Serum cholesterol level, clotting time and fibrinolytic activity	↓ Serum cholesterol ↑ fibrinolytic activity, clotting time
Chutani and Bordia (11)—India	RG = 10, fried garlic = 10	47.9 (mean)	m	0.5 g/KG BW/d	Single dose	Fibrinolytic activity (acute)	↑ fibrinolytic activity
	RG = 10, fried garlic = 10, control = 10	50.3 (mean)	m	0.5 g/KG BW/d	4 weeks	Fibrinolytic activity (long-term)	
Bhushan et al. (9)—India	RG = 15, control = 10	18–35	m	10 g/d	2 months	Blood cholesterol level	↓ Serum cholesterol
Mirunalini et al. (7)—India	RG = 20, control = 20	40–60	m	3 × 1.2 g/d	30 days	Hyperglycemia, type 2 diabetes mellitus	↓ blood glucose, TC, TG, LDL ↑ HDL, SOD, CAT, GPx,
Aslani et el. (8)—Iran	27	30–60	m/f	20 g/d	8 weeks	Lipid profile and cardiovascular risk factors	↓ BMI, SBP, SBP, TC, TG, LDL, fibrinogen ↑ HDL (within group)

AHR, aryl hydrocarbon receptor; ARNT, aryl hydrocarbon receptor nuclear translocator; ASPI, arachidonic acid-induced aggregation; BMI, body mass index; BP, benzo[a]pyrene; BW, body weight; CAT, catalase; COLL, collagen-induced aggregation; DPB, diastolic blood pressure; FBG, fasting blood glucose; GPx, glutathione peroxidase; HDL, high density lipoprotein; HIF1A, hypoxia-inducible factor 1a; JUN, proto-oncogene c-Jun; LDL, low density lipoprotein; MA, thrombelastographic maximum amplitude; NAT, N-acetyl-transferase; NFAM1, nuclear factor activated T cells activating protein with immunoreceptor tyrosine-based activation motif 1; OSM, oncostatin M; PFA, Platelet Function Analyzer PFA-100; REL, V-rel avian reticuloendotheliosis viral oncogene homolog; RG, raw garlic; SBP, systolic blood pressure; SOD, superoxide dismutase; TC, total cholesterol; TG, tiglycerides; WC, waist circumfence.

The heterogeneity regarding dosage, participants, duration of intervention and outcomes highlight the need for further well-designed clinical trials investigating potential health effects of raw garlic. In addition, drawing conclusive insights is challenging, especially considering the limited number of studies addressing various disease conditions.

## 4 Observational studies

Table 2 summarizes the results and study design of the included observational trials; two trials were prospective cohorts (20, 25), four were case–control studies (21–23, 26) and four were cross-sectional (19, 24, 27, 28). All observational studies included both male and female participants, used food frequency questionnaires (FFQ) for dietary intake assessment of raw garlic and reported beneficial effects of raw garlic consumption (19–28). Sample sizes varied among the studies (from 865 to 28,958 participants) and one study investigated not only the consumption of raw garlic, but the intake of raw garlic and raw onion taken together (25). Notably, nine out of the 10 examined trials were conducted in China (19–24, 26–28). However, the identified observational studies included diverse outcomes, such as different types of cancer (21–23, 26), prehypertension (28), newly diagnosed nonalcoholic fatty liver disease (NAFLD) (27), insulin homeostasis (25), thickened carotid intima-media thickness (cIMT) (24), risk of depressive symptoms (20) and handgrip strength (19). The follow-up time of the prospective trial investigating the risk for depressive symptoms ranged between 1 to 5 years with a median of 2 years (20), and the other prospective cohort examining insulin homeostasis conducted in the scope of the Tehran Lipid and Glucose Study (TLGS) had a median follow-up of 3 years (25). All cross-sectional trials (19, 24, 27, 28) and one prospective study (20) used the data from the Tianjin Chronic Low-grade Systemic Inflammation and Health (TCLSIH) cohort study. To identify cases, two case–control trials used county cancer registries (22, 23), one analyzed data from the Jiangsu Four Cancers Study (21) and one recruited cases from a hospital (26).

**Table 2:** Observational studies investigating the effects of raw garlic on human health.

Reference	Sample size	Age (years)	Sex	Dietary intake assessment	Outcome	Study design	Results
Zhang et al. (28)—China	22,812	39.4 (mean)	m/f	FFQ	Prehypertension	Cross-sectional	RG intake was inversely associated with prehypertension, OR (4 times/week to 1 time/day) = 0.96 (0.87, 1.06), OR (≥ 2 times/day) = 0.69 (0.52, 0.90) (*p* for trend = 0.06)
Gu et al. (19)—China	28,958	≥ 18	m/f	FFQ	Handgrip strength	Cross-sectional	Handgrip strength is related to RG intake, males: OR (≥ 2–3 times/week) = 0.66 (0.58, 0.74), females: OR (≥ 2–3 times/week) = 0.77 (0.69, 0.87)
Zhang et al. (27)—China	24,106	20–90	m/f	FFQ	Newly diagnosed NAFLD	Cross-sectional	OR for NAFLD associated with each 1 g of RG/1000 kcal: 0.93 (0.90, 0.97) in men
Liu et al. (23)—China	9,944	≥ 18	m/f	FFQ	Liver cancer risk	Case–control	RG consumption ≥2 times/day is inversely associated with liver cancer, OR = 0.77 (0.62, 0.96)
Jin et al. (21)—China	10,988	≥ 18	m/f	FFQ	Interactions with tobacco smoking, alcohol drinking and esophageal cancer	Case–control	RG consumption is inversely associated with esophageal cancer, OR (≥ 1 time/week) = 0.68 (0.57, 0.80)
Myneni et al. (26)—China	865	≥ 20	m/f	FFQ	Lung cancer risk	Case–control	RG consumption is associated with lower lung cancer risk, OR (≥ 2 times/week) = 0.50 (0.34, 0.74)
Jin et al. (22)—China	5,967	≥ 18	m/f	FFQ	Lung cancer risk	Case–control	RG intake ≥2 times per week is inversely associated with lung cancer: OR = 0.56 (0.44, 0.72)
Mirmiran et al. (25)—Iran	1,141	≥ 18	m/f	FFQ	Insulin homeostasis	Prospective cohort	RG and raw onion intake is associated with decreased risk of insulin resistance: OR = 0.61 (0.38, 1.00), and hyperinsulinemia: OR = 0.59 (0.36, 0.96)
Liu et al. (24)—China	4,329	≥ 19	m/f	FFQ	Thickened carotid intima thickness	Cross-sectional	Light to moderate RG intake is inversely associated with thickened carotid IMT: OR (2–3 times/week) = 0.71 (0.55, 0.92)
Wang et al. (20)—China	7,427	39.7 (mean)	m/f	FFQ	Depressive symptoms	Prospective cohort	RG consumption is associated with reduced risk of depressive symptoms in females: OR (2–3 times/week) = 0.72 (0.54, 0.97)

FFQ, food frequency questionnaire; IMT, intima-media thickness; NAFLD, nonalcoholic fatty liver disease; OR, odds ratio; RG, raw garlic.

The prospective studies reported, that consumption of raw garlic 2–3 times/week was associated with a lower risk of depressive symptoms in females (odds ratio (OR) 0.72 [95% confidence interval 0.54, 0.97]) (20). Moreover, raw garlic and onion intake was associated with decreased risk of insulin resistance: OR = 0.61 (0.38, 1.00), and hyperinsulinemia: OR = 0.59 (0.36, 0.96) (25). The cross-sectional studies also observed favorable findings regarding their outcomes. Raw garlic intake was inversely associated with prehypertension, with those consuming raw garlic at least twice daily exhibiting a 31% lower odds for prehypertension (OR = 0.69, CI: 0.52–0.90), suggesting a possible protective effect (28). Moreover, high intake of raw garlic was associated with higher handgrip strength, both evident in males (OR = 0.66, CI: 0.58–0.74) and females (OR = 0.77, CI: 0.69–0.87) (19). Furthermore, each additional gram of raw garlic per 1,000 kcal was associated with reduced odds of newly diagnosed NAFLD in men (OR = 0.93, CI: 0.90–0.97) (27). Moreover, raw garlic consumption was inversely associated with thickened cIMT (OR = 0.74, CI: 0.59, 0.94 for 1 time/week and OR = 0.71, CI: 0.55, 0.92 for 2–3 times/week) (24). Lastly, the included case–control trials provided evidence on the potential cancer-related health benefits of raw garlic consumption. Raw garlic consumption of at least twice daily was associated with a lower odds of liver cancer (OR = 0.77, CI: 0.62–0.96) (23), while consuming garlic at least once weekly was associated with a lower odds of esophageal cancer (OR = 0.68, CI: 0.57–0.80) (21). Furthermore, two trials concluded that raw garlic intake at least twice per week was associated with lower lung cancer risk: OR = 0.50, CI: 0.34–0.74 (26) and OR = 0.56, 0.44, 0.72 (22).

## 5 Discussion

In the present mini review, we identified 12 clinical trials and 10 observational studies on the health effects of raw white garlic consumption. The clinical trials indicated improvements in (cardio)metabolic biomarkers, fibrinolytic activity, and anthropometric measures. The identified observational studies suggested that raw garlic intake had beneficial effects on prehypertension, handgrip strength, risk of different types of cancer, thickened cIMT, insulin homeostasis, and newly diagnosed NAFLD.

The potential benefits for human health observed in intervention and observational studies can be explained by a range of mechanisms. Garlic bulbs contain around 2.3% organic sulfur compounds, with alliin comprising 80% of cysteine sulfoxide (29). When garlic is crushed, these compounds react with alliinase to form allicin, making up 70–80% of resulting thiosulfinates, which are unstable in nature and quickly decompose into various sulfur compounds like diallyl sulfide diallyl disulfide, diallyl tetrasulfide, diallyl trisulfide, ajoene and further compounds (5, 29).

The cardiovascular protection primarily revolve around reducing lipid levels, attenuating oxidative stress, antiplatelet effects, inhibiting angiogenesis, safeguarding the endothelial cell layer, reducing inflammation, and these processes are mediated through various signaling pathways (such as AMPK/TLRs, GEF-H1/RhoA/Rac, PPARγ/LXRα, Keap1/Nrf2 and PI3K/AKT) as described by Li and colleagues (30). The main antilipemic effect is attributed to the inhibition of cholesterol synthesis by inhibiting the activity of hydroxy-3-methyl glutaryl coenzyme A reductase (HMG-CoA), a key enzyme in cholesterol synthesis (30) and by blocking CD36 expression and oxidized LDL (oxLDL) uptake in human macrophages through the PPARγ pathway, potentially preventing atherosclerotic lesions (31, 32). Garlic compounds may exert their antioxidative effects mainly through enhancing levels and/or activity of SOD, CAT and GPx (6, 32), scavenging free radicals and attenuating lipid peroxidation (6). Garlic can have antithrombotic effects by inhibiting cyclooxygenase-mediated thromboxane synthesis (6) and by interacting with the biosynthesis of prostaglandins via cyclooxygenase 1 inhibition and with fibrinogen receptors (15). To summarize, antiplatelet activity of garlic compounds may come from various pathways, including the reduced synthesis of prothrombotic factors like cyclooxygenase-1, thromboxanes, leukotrienes, prostaglandins, and a reduced secretion of arachidonic acid from phospholipids and coagulation factor IV from platelets (30). Additionally, garlic’s ability to inhibit platelet aggregation induced by calcium ion aggregates may further contribute to its antiplatelet effects (30).

Proposed anticancer effects of garlic compounds may be based on a wider range of mechanisms, such as altering mitochondrial permeability, inhibiting angiogenesis and invasion, scavenging free radicals and preventing the formation of DNA adducts, activating enzymes for carcinogen detoxification, regulating cell proliferation and apoptosis, maintaining chromosome stability and immune response, modifying histones, inhibiting carcinogenic activation, and altering protein degradation dependence on the proteasome (6, 29).

Garlic and its extracts have no known toxic compounds (6). In rats, administration in high doses of garlic powder (500 mg/kg) resulted in changes in lung and liver tissue, indicating dose-related toxicity, but low doses (50 mg/kg) had little effect (33). Long-term supplementation of high doses of fresh garlic homogenate (1,000 mg/kg per day) in rats caused significant reductions in endogenous antioxidants (catalase and SOD) without altering lipid peroxidation levels and the animals treated with this dose showed morphological changes in the liver, indicating liver injury (34). This study also found that garlic in low doses had the potential to enhance the antioxidant status, but at higher doses a reversal of these effects was observed (6, 34). In humans, garlic is generally considered safe by regulatory authorities, but can cause gastric irritation and different side effects, including gastrointestinal discomfort and body odor, especially at high doses (6). Thus, the acceptability of higher doses of raw garlic needs to be considered in future intervention studies.

While recognizing the constraints of the present study, the findings should be approached with caution due to the necessity for further standardized methods in future research on this topic to assure the reliability of the trials included. Some studies exhibited small sample sizes and often had short durations, which is why generalizability remains uncertain. Also, the epidemiological investigations predominantly originated from China, underlining the necessity for additional epidemiological data from different locations to spot potential geographical variations, if any. In the meantime, it is essential to acknowledge the health-promoting findings from these studies and the valuable data they provide, indicating promising trends in improving human health. If future research supports these results, raw garlic may become a potential contributor to dietary guidelines aimed at enhancing human health.

## 6 Future perspectives

In conclusion, the lack of clinical studies specifically dedicated to raw white garlic represents a significant gap in the understanding of its health benefits - despite its affordability and widespread availability - and hinders the development of evidence-based dietary recommendations on garlic consumption. The reliance on research involving aged black garlic and garlic extracts limits the generalizability of findings to the widely consumed raw white garlic. The gap in research is evident given the distinct chemical composition of raw garlic compared to its aged counterparts or extracts and existing studies often lack unity in garlic preparation, dosage, and duration, making it challenging to draw meaningful conclusions about the unique properties of garlic (5, 35). To fully appreciate and utilize the potential health benefits of this easily accessible vegetable, research should prioritize and conduct well planned clinical trials focused on raw garlic, exploring its unique properties and applications in promoting human health.
